# Visualization and Cybersecurity in the Metaverse: A Survey

**DOI:** 10.3390/jimaging9010011

**Published:** 2022-12-31

**Authors:** Yang-Wai Chow, Willy Susilo, Yannan Li, Nan Li, Chau Nguyen

**Affiliations:** Institute of Cybersecurity and Cryptology, School of Computing and Information Technology, University of Wollongong, Wollongong, NSW 2522, Australia

**Keywords:** augmented reality, cybersecurity, extended reality, Metaverse, visualization, virtual reality

## Abstract

The popularity of the Metaverse has rapidly increased in recent years. However, despite the attention, investment, and promise of the Metaverse, there are various cybersecurity issues that must be addressed before the Metaverse can truly be adopted in practice for serious applications. The realization of the Metaverse is envisioned by many as requiring the use of visualization technologies such as Virtual Reality (VR) and Augmented Reality (AR). This visual aspect of the Metaverse will undoubtedly give rise to emerging cybersecurity threats that have not received much attention. As such, the purpose of this survey is to investigate cybersecurity threats faced by the Metaverse in relation to visualization technologies. Furthermore, this paper discusses existing work and open research directions on the development of countermeasures against such threats. As the Metaverse is a multidisciplinary topic, the intention of this work is to provide a background of the field to aid researchers in related areas.

## 1. Introduction

The Metaverse is widely anticipated to be the next evolution of the Internet [1]. Furthermore, the development of Web 3.0 with its decentralized nature is seen as being complementary to the progress of the Metaverse. In fact, Gartner [2] predicts that by as early as the year 2026, 25% of people will spend at least an hour a day in the Metaverse. While there is no universal definition for the Metaverse [3], it is broadly seen as a collective virtual space that is shared by many users through the Internet and is created through the convergence of physically persistent virtual space and virtually enhanced physical reality. It is a shared virtual space where users, represented by digital avatars, can communicate, collaborate, and socialize with each other and interact with digital things in computer-generated virtual worlds [4].

The term “Metaverse” is a portmanteau of “meta”, which is Greek for transcendence, and “verse”, which is from the word universe. It is a term that originated 30 years ago from the science fiction novel *Snow Crash* by Neal Stephenson in 1992, who wrote of humans who physically live in the real world but mentally spend much of their time in a three-dimensional (3D) parallel virtual world, called the *Metaverse*, using personal computer terminals with pictures projected onto goggles [4,5]. Within the Metaverse, people appear in the form of personalized digital avatars where they can communicate with others and perform activities that mimic real life within the virtual world.

While the development of the Metaverse is still in its infancy, there are a number of digital virtual worlds that can be seen as early precursors of the Metaverse. Some of the earliest examples can be found in the form of text-based multi-user dungeons (MUD) games that emerged in the late 1970s [1]. This was followed by the evolution of online 3D virtual worlds, such as the virtual social world named *Second Life*, and virtual game worlds such as the popular massively multiplayer online role playing game (MMORPG), the *World of Warcraft*, which have their own currencies and digital economies [5].

With advances in technologies, e.g., 5G, extended reality (XR), artificial intelligence (AI), and blockchain, required to make the Metaverse a reality, many now see the feasibility of building Metaverse applications and the prospects that they bring. This has attracted the interest of many large tech companies, including Facebook (now renamed “Meta” in view of the Metaverse), Microsoft, Apple, and Nvidia, which are investing in the development of various Metaverse platforms. For example, Meta has its immersive workrooms platform known as *Horizon Workrooms* [6], Microsoft has a similar collaborative platform for live virtual events called *AltspaceVR* [7], and Nvidia has its *Omniverse* [8] platform for creating Metaverse applications.

Despite the attention, investment, and promise of the Metaverse, there are a variety of cybersecurity issues that must be addressed before the Metaverse can truly be used in practice for any serious applications. However, this is complicated by the fact that the development of the Metaverse is still in its early stages, and its realization requires the integration of many different technologies. The combination of various technologies will create a large cyber attack surface, which will undoubtedly result in many new cybersecurity issues. In addition, given the widely accepted visual nature of the Metaverse that relies on visualization technologies such as virtual reality (VR) and augmented reality (AR), this visual aspect will give rise to emerging cybersecurity threats. For example, researchers have shown that VR devices are vulnerable to inference attacks that can reveal private information [9,10], a victim’s AR device can be tracked in real-time thereby compromising location privacy [11], and attackers can cause physical harm through attacks that induce cybersickness and user disorientation [12,13].

The purpose of this paper is to investigate cybersecurity issues faced by the Metaverse in relation to visualization technologies. Unlike other more traditional cybersecurity domains, e.g., networking and data protection, the security of visualization technologies is an area that has not received much attention to date. However, defenses against cybersecurity threats in the visual domain will become increasingly more important as the development of the Metaverse becomes more mature. This is the motivation behind this paper.

The main contributions of this work are summarized as follows. This survey presents the following:

- An investigation of cybersecurity issues, in particular, cyber threats faced by the Metaverse in relation to the use of visualization technologies;
- A discussion of existing work and open research directions on the development of countermeasures against such threats.

As the development of the Metaverse encompasses many different disciplinary areas, the intention of this work is to provide a background of the field to aid researchers in related areas.

The rest of this paper is organized as follows. Section 2 discusses related work and how they differ from the work in this paper. Next, Section 3 presents an overview of Metaverse and visualization technologies. This is followed, in Section 4, by a survey of the cybersecurity issues faced by the Metaverse in relation to visualization. Section 5 and Section 6 discuss potential countermeasures and promising open research directions, respectively. This paper then concludes in Section 7.

## 2. Related Work

With the popularity of the Metaverse, there are a number of surveys in the literature that have reviewed research efforts focused on different facets of the Metaverse. The approach adopted in this survey was to search major scientific databases, namely, IEEE Xplore, the ACM digital library, Google Scholar, and Scopus, for research papers related to the cybersecurity of VR and AR systems and the Metaverse. The inclusion criteria were mainly papers that were published within the last five years and specifically focused on the technical and social aspects of cybersecurity related to the usage of visualization technologies in virtual worlds. This section presents a summary of recent surveys that look at various technical aspects of the Metaverse and discusses how the work in this paper differs from other surveys. Table 1 presents a summary of recent surveys on the Metaverse and related technologies.

From Table 1, it can be seen that some existing surveys discuss general aspects of the Metaverse. Recent work by Cheng et al. [1] described aspects of the Metaverse that have been heavily advocated by the industry and the outlooks of several major tech companies. Their work also discussed the authors’ vision of what the key technical requirements of the Metaverse should be, along with an overview of existing social VR platforms. On the other hand, Park and Kim [3] presented a survey of the Metaverse from a different perspective. In their work, they discussed the essential concepts and necessary techniques for realizing the Metaverse in terms of hardware, software, and content. They also analyzed Metaverse approaches to user interaction, implementation, and application from the viewpoint of existing representative Metaverse applications. This work also discussed the limitations and open challenges of implementing an immersive Metaverse.

Other surveys investigated the Metaverse in relation to specific technologies. For example, Yang et al. [14] discussed how blockchain and AI technologies can be fused with the Metaverse by presenting a survey investigating state-of-the-art studies of major Metaverse technologies, including digital currencies, AI applications in the virtual world, and blockchain-empowered technologies. In other work on blockchain, Huang et al. [15] examined how building information modeling and blockchain technologies can be integrated with the Metaverse, while Fu et al. [16] reviewed the role of blockchain and intelligent networking in providing immersive Metaverse experiences. In terms of AI technologies, Huynh-The et al. [17] presented a survey that investigated the role of AI and its integration in the development of the Metaverse. They also examined the potential of AI-based methods in building virtual worlds for the Metaverse.

There have also been several recent surveys focused on investigating various security issues faced by the Metaverse. For instance, Wang et al. [4] conducted a comprehensive survey on the fundamentals of the Metaverse, such as its characteristics and general architecture, as well as the security and privacy threats faced by the Metaverse. In their work, they described various categories of security threats and the critical challenges encountered by different aspects of the Metaverse, along with existing research on countermeasures against these threats. Their work also discussed potential solutions and future research directions for building a secure Metaverse. Other surveys on security and the Metaverse include the work by Fernandez and Hui [18] on privacy, governance, and ethical design in the development of the Metaverse, and a survey by Di Pietro and Cresci [19] that discussed several security and privacy issues, and risks in the context of the Metaverse.

There are also several other surveys that are not focused specifically on the Metaverse but on its related technologies. This includes the taxonomy of cybersecurity challenges faced in VR environments presented in Odeleye et al. [20], where they systemically classify existing VR cybersecurity threats against existing defenses; the work by Böhm et al. [21], who conducted a structured literature review on the combined use of AR with digital twin technology in the context of cybersecurity and discussed the benefits and security-related application areas for these combined technologies; and the survey by De Guzman et al. [22] that reviewed various protection methods that have been proposed to ensure user and data security and privacy in mixed reality (MR). Other related work includes a survey on edge computing with digital twin technology [23] and a Metaverse digital twin resource allocation framework [24].

From the overview of related work presented above, it can be seen that existing surveys do not directly address visualization and cybersecurity in the context of the Metaverse. This paper intends to bridge this gap by investigating cybersecurity issues and countermeasures that are specifically focused on the context of visualization technologies and their use in the Metaverse. In this paper, visualization technologies refer to technologies that are required to present a user with a visual representation of the Metaverse and that allow the user to interact with this visual information.

## 3. Background

There is a multitude of components that are required to realize the Metaverse. This section provides a brief overview of the main technologies of the Metaverse. This will be followed by a discussion of visualization technologies, which is the focus of this survey.

### 3.1. Metaverse Technologies

**Figure 1:**
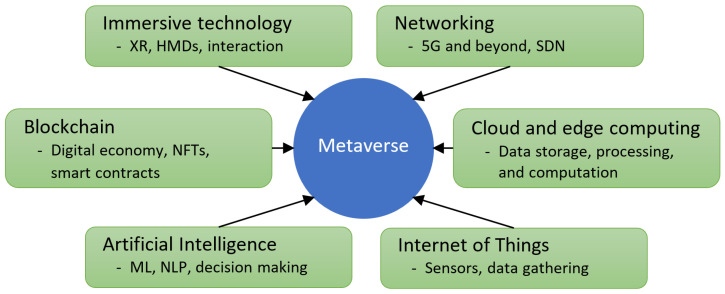
Key technologies required to empower the Metaverse.

Figure 1 depicts several major technologies required to empower the Metaverse. Immersive technologies are one of the key components of the Metaverse. These technologies are vital for connecting humans to the virtual world and allowing them to interact with virtual content using interaction devices, e.g., handheld controllers, or hands-free gesture-based methods. Immersive technologies encompass visualization technologies, such as extended reality (XR) and Head Mounted Displays (HMDs), to present the user with a visual representation of virtual content in the Metaverse.

Networking is essential for communication and data transmission over the Internet. This is required for connecting Metaverse users from all over the globe. Advances in 5G technology and beyond offer more efficient and reliable means of connectivity, which will empower the Metaverse. Software-defined networking (SDN) is a technology that enables dynamic network management by separating the control plane from the data plane. It is seen as a promising future direction in the networking field.

Artificial intelligence (AI) technology will be central to running the Metaverse and in automating a variety of processes. There is a wide range of AI technologies, including machine learning (ML), deep learning, natural language processing (NLP), computer vision, and so on. This technology will be used in the Metaverse for various purposes such as for virtual world content generation, scene understanding, object detection, speech-to-text and text-to-speech processing, and human action/activity recognition [17].

Cloud and edge computing will be required for distributed data storage and efficient computation and processing of the vast amount of Metaverse data. The decentralized nature of blockchain technology will be vital for enabling the digital economy in the Metaverse through things such as non-fungible tokens (NFTs) and smart contracts. The Internet of Things (IoT) will be essential for connecting the Metaverse to the real world, for example, by obtaining data from the real world through various sensors and IoT devices. The collected data will be infused into the Metaverse to give rise to the convergence of physically persistent virtual space and virtually enhanced physical reality.

### 3.2. Visualization Technologies

There are a variety of different definitions of the Metaverse that can be found in the literature [3]. The general definition of the Metaverse as a collective virtual space that is shared by many users does not explicitly require the Metaverse to adopt visualization technologies [3]. Nevertheless, many deem visualization to be a key component of the Metaverse [1,3,4,5,17]. In fact, companies such as Meta consider VR to be the foundation to build the Metaverse and have heavily invested in the development of this technology [1]. This can also be seen in the platforms developed by the major tech companies, e.g., *Horizon Workrooms* [6], *AltspaceVR* [7], and *Omniverse* [8].

**Figure 2:**
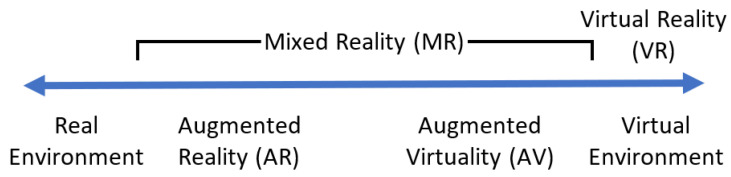
The reality–virtuality continuum introduced by Milgram and Kishino [25].

In terms of visualization, many people associate extended reality, often abbreviated as XR, with the Metaverse. XR is in fact an umbrella term that encompasses augmented reality (AR), virtual reality (VR), mixed reality (MR), and other immersive technologies. To understand the differences between AR, VR, and MR, Figure 2 illustrates the reality-virtuality continuum introduced by Milgram and Kishino [25], in which the real world is located at one end of the continuum while the virtual encironment is at the other end. In the reality–virtuality continuum, MR is defined as an environment that blends real and virtual content. AR is where the real environment is augmented with virtual content, i.e., virtual content is superimposed onto the real world. On the other hand, VR is where the user is immersed in completely virtual computer-generated content and has a limited perception of the real environment. As such, VR is located towards the right-hand side of the reality–virtuality continuum.

Immersing the user in the Metaverse using XR typically relies on the use of head-mounted displays (HMDs). An HMD is a wearable display device that is worn by the user, where images representing virtual content are projected to the user’s visual system through the HMD’s built-in displays. At the time of writing, some of the commonly used HMDs include Meta’s Quest 2 [26], Microsoft’s HoloLens 2 [27], the HTC VIVE Pro 2 [28], and the Valve Index [29]. HMDs are often equipped or coupled with a tracking mechanism to track user head movement to update images in real- time based on where the user is looking. If the images are not updated and displayed to the user fast enough, this can induce cybersickness, which can result in various adverse physical effects, e.g., eyestrain, headache, disorientation, and nausea [30]. In addition, HMDs often come with a pair of handheld controllers for users to interact with virtual content.

## 4. Visualization and Cybersecurity Issues

As previously discussed, the realization of the Metaverse requires a combination of multiple technologies, which presents adversaries with a large cybersecurity attack surface. Table 2 presents an overview of the visualization and cybersecurity issues and potential countermeasures. This section examines various cybersecurity issues and threats associated with the use of visualization technologies, while potential countermeasures are discussed in Section 5.

### 4.1. Authentication and Identity

As the Metaverse is a virtual space where users interact with other users and with the virtual environment through digital avatars. A digital avatar is a virtual representation of a user in the virtual world. Hence, authentication in the Metaverse is vital for ensuring that a user is legitimate and for verifying that the person is who they claim to be. It is also imperative to safeguard a user’s identity and authentication credentials to prevent identity theft and impersonation attacks.

The traditional means of authentication often rely on a username and password combination. When this concept is transferred to the Metaverse setting, a user is typically presented with a virtual keyboard, where they either air-tap using hand gestures or use a controller to point-and-click on virtual keys. Methods such as multifactor authentication using a device such as a smartphone are cumbersome if a user is wearing an HMD and holding handheld controllers. This is because a VR HMD deliberately blocks off the user’s view of the real world. If authentication requires the use of a smartphone, the user will have to put down the handheld controllers and remove the HMD before being able to authenticate with the smartphone. After using the smartphone, the user will have to put on the HMD and take up the handheld controllers again.

The virtual keyboard method of authentication is vulnerable to keystroke inference attacks, as there are various methods for inferring virtual keystrokes. This includes methods such as wireless signal-based attacks, video-based attacks, and malware-based attacks. For example, Al Arafat et al. [9] developed VR-Spy, which uses side-channel information in the form of WiFi signals to recognize virtual keystrokes in VR headsets. In other work, researchers showed that hand gesture patterns can be exploited to infer the keystrokes when a user is required to enter virtual keyboard input via air-tapping [10]. Others have shown that computer vision methods can be used to infer input when a user uses a point-and-click device, and information extracted from the motion sensors of a pointing device can also be used to infer input [31]. In addition, Lou et al. [32] demonstrated malware-based keystroke inference attacks on an MR device. The vulnerabilities of swipe pattern-based authentication in VR have also been studied [33].

If a person’s authentication credentials are compromised, an adversary can commit identity theft and impersonation attacks by stealing a legitimate person’s identity and impersonating that person to fool a victim in the Metaverse into believing that they are interacting with that person. This can easily be achieved in the Metaverse because people interact through digital avatars. Moreover, with advances in AI and computer vision for processing and generating facial data, DeepFake technology has recently attracted widespread attention. Deepfake technology uses deep learning tools to manipulate images and videos and is often used to swap faces with other faces [40]. Bose and Aarabi [41] showed that DeepFake techniques can be used in VR to replace a user’s face with another face. Using this technology, an adversary can impersonate another person to conduct a transaction or spread disinformation to deliberately cause reputation and psychological damage.

### 4.2. Privacy Issues

Issues concerning user privacy are rife on the Internet today and will only be exacerbated by the Metaverse. Privacy not only is restricted to a user’s digital identity but also includes other sensitive information such as a user’s digital footprint. Falchuk et al. [47] breakdown privacy into the privacy of personal information, the privacy of behavior, and the privacy of communications. They define personal information as referring to anything that reveals physical, medical, physiological, economic, cultural, or social status; behavior refers to information about habits, activities, choices, etc.; and communications relates to data associated with personal communications.

Metaverse applications are real-time applications that require the frequent and timely exchange of data between end-user devices and servers. This exposes users to various cybersecurity threats, such as the leakage of private location information. In the work by Shang et al. [11], they developed an automated user location tracking system for multiuser AR applications called ARSpy. Using this system, they demonstrated that their attacks could accurately track a victim solely based on the victim’s network traffic information. This makes their attack difficult to detect and allows an adversary to covertly discover the location of an AR device and to track the user’s physical location in real-time.

In other work, Yarramreddy et al. [48] explored the forensics of immersive VR systems and their social applications. They demonstrated that a significant amount of data, such as user identities, network artifacts, and events, can be extracted from the systems. This potentially leaks a significant amount of private information that can be exploited by adversaries. For example, an attacker can hijack or eavesdrop on user sessions. They also found that attacks such as Man-In-The-Middle (MITM) were plausible due to the lack of encryption, where an attacker can potentially inject themselves into a private room in the virtual world without the need for further authentication. In addition, Vondrek et al. [49] also demonstrated how malware could be used to conduct man-in-the-room attacks in VR systems.

Another potential privacy attack can exploit the equipment used for visualization. The majority of XR HMDs are now equipped with front-facing cameras. These are used for movement tracking or for capturing the video feed from the real world, e.g., in an AR application, virtual content can be superimposed on video images before they are displayed to the user. This video feed can be streamed to an attacker, which will allow the attacker to see the real world around the victim [12,51]. This can reveal a variety of personal information, such as where the victim is located, who they are with, private content in their room, and so on.

### 4.3. Social Issues

The Metaverse is a shared virtual space that transcends physical limitations. The rules and controls of how to interact with and navigate in this virtual space are designed by the application developers. If appropriate mechanisms are not in place, this can allow for virtual stalking and/or spying. For example, in the social virtual world of *Second Life*, users can use a virtual camera as a spying device by placing it at a certain location to observe other avatars and their interactions [52]. An adversary can even virtually stalk others by attaching a virtual camera to another avatar without the victim being aware of it. These are things that are not easily achieved in the real world due to laws and physical limitations but are effortless in the virtual world if the system permits it and no restrictions or regulations are in place.

Virtual harassment and other forms of online abuse are common social issues that occur particularly in shared online environments. The Metaverse, unfortunately, comes with its fair share of unwanted social behavior, and there are many stories of virtual harassment in VR [55,56,57]. Visualization technologies aim to immerse users in the Metaverse and to create a sense of presence, i.e., the sense of “being there”. However, this embodiment and presence make harassment feel more intense [58]. Furthermore, the 3D visual nature of VR gives rise to violations of personal space such as simulated touching or grabbing. In a study by Blackwell et al. [58], they found that affordances of VR, compounded with features such as synchronous voice chat, exacerbate abuse. They also reported that, given non-standardized application controls, it is difficult to escape from or report unwanted behavior. Moreover, it is difficult to regulate such virtual spaces because what constitutes online harassment is often subjective and highly personal.

### 4.4. Physical Threats

XR technologies are immersive systems that place the human-in-the-loop. This is because the user’s senses, e.g., visual and auditory, are presented with up-to-date information from the system, so that the user can interact with the virtual content, which in turn is updated by the system. Thus, forming a feedback loop. This gives rise to the possibility of immersive attacks. Immersive attacks are attacks in which the virtual environment is maliciously modified with the intention to cause physical or mental harm or to disrupt the user [12]. In their work on immersive attacks, Casey et al. [12] demonstrated a number of proof-of-concept attacks in a VR system.

When a user wears a VR HMD, the user’s visual perception is intentionally blocked off from the real world to create an immersive experience. As such, before using a VR system, some systems allow the user to define virtual boundaries, referred to as the Chaperone, to prevent the user from injuring themselves, e.g., by knocking into real-world objects or by walking into a real wall. In a Chaperone attack, the boundaries are deliberately modified, which can result in the user physically injuring themselves [12]. A disorientation attack is one that deliberately elicits a sense of dizziness and confusion from the user. Casey et al. [12] also coined the term human joystick attack to refer to an attack used to manipulate the user’s physical movement to a predefined location without the user realizing it, whereas an overlay attack is one where an image is deliberately displayed to block the user’s view of the virtual environment.

By hijacking a VR system, an attacker can also launch physical attacks such as causing light flashes to induce epilepsy or making the audio excessively loud to cause hearing loss. Odeleye et al. [13] demonstrated attacks that can result in cybersickness by manipulating GPU frame rate and network vulnerabilities. In a GPU-based attack, they used malware to intentionally overwhelm GPU resources to disrupt the rendering frame rate. While in a network-based attack, they used a script to launch a ping flooding attack to disrupt network traffic in a collaborative VR environment. This resulted in scene-tearing artifacts in the graphics and a drop in the frame rate. Disrupting the rendering frame rate increases the latency of images presented to the user, which will adversely affect user experience and likely induce cybersickness.

In other work on physical threats in XR systems, Tseng et al. [61] defined virtual–physical perceptual manipulations that they divided into two main classes, which they called puppetry attacks and mismatching attacks. Puppetry attacks are attacks aimed at controlling the physical actions of different body parts of an immersed user, while mismatching attacks are where an adversary exploits a misalignment of information between a virtual object and its physical counterpart to cause physical harm. In their work, they presented various scenarios and demonstrated how such attacks could potentially be applied in practice.

## 5. Countermeasures

The previous section highlighted various cybersecurity issues in relation to visualization that are faced by the Metaverse. To address these issues, researchers have worked on potential solutions to overcome them. This section discusses existing work on the development of defenses and countermeasures against cybersecurity threats in the Metaverse.

### 5.1. XR Authentication

Given the increasing popularity of XR HMDs, it is becoming increasingly important to design secure and usable user authentication methods for these systems. Unlike traditional keyboard, mouse, or touchscreen interaction methods, XR users typically use gestures, via hands-free means or a handheld controller, to perform input. While gestures can be used to enter text passwords or personal identification numbers (PINs), it is cumbersome and vulnerable to shoulder-surfing attacks by an external observer [34] or keystroke inference attacks [9,10,31,32]. Moreover, it is impractical to require a user to unequip an HMD, perform authentication, and then put the HMD back on. As such, alternative secure authentication methods are required for XR systems.

Examples of alternative authentication methods include RubikAuth which was proposed by Mathis et al. [35]. RubikAuth is a 3D authentication method in VR, inspired by a 3D Rubik’s cube, which is resilient to shoulder-surfing attacks. It was designed for point-and-click type input devices, where a user is presented with a 3D cube with digits and colors and the user can rotate the 3D cube to change its orientation. User authentication is performed by selecting color–digit combinations from the cube using either eye gazing, head pose, or tapping with a controller. In other work, Abdelrahman et al. [36] presented CueVR, a cue-based authentication method in VR, to avoid shoulder-surfing attacks by requiring authentication through visual cues presented to the user in a VR HMD. Both RubikAuth and CueVR are PIN-based authentication methods.

Instead of using passwords or PINs, there is another line of research that utilizes user behavior or biometric characteristics for authentication. For instance, Kupin et al. [37] and Pfeuffer et al. [38] investigated behavioral biometrics in VR, which is based on the concept that every person has uniquely individual behavioral characteristics. This is a form of continuous authentication that does not require explicit authentication, e.g., entering a password, as a user’s identity is continuously assessed in the background based on their behavioral patterns. A similar method of biometric user identification from kinesiological movements, which are unique for each person, was proposed by Olade et al. [39].

### 5.2. AI-Driven Cybersecurity

AI-driven cybersecurity techniques will become increasingly important in detecting and preventing malicious activity in the Metaverse. Techniques such as machine learning can be used to detect abnormal behavior and warn users of potential attacks. For example, Odeleye et al. [13] showed that a machine learning method can be used to provide a warning against GPU frame rate manipulation and network vulnerabilities that can cause cybersickness. These techniques can also be used to monitor XR systems for abnormal activity such as the kind of manipulations used to execute immersive attacks, e.g., altering boundaries of the Chaperone and subtle changes to the virtual environment or the user’s visual cues [12]. Furthermore, AI-driven cybersecurity techniques can also potentially be used to detect malware in XR systems, social misbehavior, virtual spying, etc.

In addition to AI techniques being used to detect suspicious activity, they can also be used to detect malicious AI-generated content. In a study by Aliman and Kester [42], they analyzed generative AI, such as DeepFakes in VR, and proposed a cybersecurity-oriented procedure to generate defenses against such unethical practices. Additionally, various methods have been developed to detect DeepFakes [40], where many of these methods rely on AI techniques themselves to detect face swapping and other identity manipulations in images and video [43,44,45,46].

### 5.3. Rules and Governance

In light of the various social issues arising from unwanted interactions among users in the Metaverse, e.g., harassment and abuse, researchers have examined a number of methods to mitigate such issues. For one thing, system developers can decide on what features to provide and rules to enforce; they can constrain what users can or cannot do, provide permissions, restrict access, and so on. Such governance and control in virtual worlds can be coded into the software by developers [18,53]. For example, Meta’s Horizon Worlds introduced personal boundaries that will enforce a distance between a user’s avatar and others to avoid unwanted interactions [54]. The distance can be adjusted and customized to different settings according to a user’s preferences.

However, things such as social behavior norms cannot be coded. While developers and companies can create rules to govern social behavior and enforce penalties for misbehavior, rules are often cumbersome to implement and potentially invasive as they often require a certain degree of monitoring [53]. As social norms and online harassment are relatively subjective, it has been suggested that community-led governance is one of the potential solutions for mitigating virtual harassment and online abuse [58]. To date, there are limited governance tools for online communities. Schneider et al. [59] propose the development of an open standard for networked governance of online communities. They propose a governance design that is dynamic in which community members can engage in the creation and experimentation of different governance techniques.

To prevent unwanted behavior such as virtual stalking and spying, Falchuk et al. [47] discussed a number of privacy strategies. For example, the creation of clones that appear to be identical to the user’s avatar can confuse observers and result in them losing track of the user’s avatar. Users can be allowed to create a private copy of a portion of the virtual environment, where only the user and other invited users can inhabit that private space. Teleport functionality can be included to allow users to be transported to a new location in the virtual world to throw an observer off track.

### 5.4. Cybersickness Mitigation

Disruptions in the update and a display of virtual content can induce cybersickness. Such disruptions in performance can be the result of various factors including high computational load, network issues, and cybersecurity attacks (e.g., denial of service (DoS)), in the Metaverse. Hence, it is vital to develop methods to mitigate the negative effects on a user’s physical well-being resulting from attacks or other disruptions.

For instance, Valluripally et al. [60] proposed a quantitative framework to analyze potential security and privacy issues that induce cybersickness in order to incorporate security design principles to mitigate their effects. Their study was based on a social VR learning environment application, where they determined that DoS attacks, data leakage, man-in-the-room attacks, and unauthorized access are the most vulnerable components that can result in higher levels of cybersickness. The severity of these threats was assessed in relation to their impact on cybersickness and degradation of application functionality. Using their framework, they demonstrated that by applying a combination of security design principles, e.g., hardening, diversity, redundancy, and the principle of least privilege, they were able to develop effective mitigation strategies. This showed that it is possible to reduce the occurrence of cybersickness by incorporating security design principles with different levels of abstraction in a virtual world and by dynamically adjusting the functionality of the system based on the cyber threat level.

### 5.5. XR Forensics

With the increasing use of XR technologies for the Metaverse, digital forensics will become increasingly important to investigate and detect malicious software or to find evidence of an attack. Casey et al. [50] conducted a study on memory forensics of immersive VR systems, which can be used to detect malware and exploitation tools influencing the system. They developed a tool for analyzing memory dumps from a VR system and showed that various pieces of information can be extracted from the memory dumps as potential sources of digital evidence. In addition, the use of forensics of immersive VR systems can also reveal vulnerabilities in the system that an adversary can exploit [48,49]. This will be useful for developers to facilitate the design of security mechanisms to safeguard their systems.

## 6. Open Research Directions

In addition to the areas discussed in the previous section, there are various open research areas on visualization and cybersecurity in the Metaverse. This section discusses some of these directions.

### 6.1. Continuous Authentication

To overcome the issues and vulnerabilities associated with the use of passwords and PINs for authentication in the Metaverse, continuous authentication is a promising open research direction. This approach not only overcomes problems such as shoulder-surfing attacks and inference attacks but also presents an attractive method of authentication, where a user does not have to remember a password/PIN. Furthermore, this non-intrusive continuous approach provides an advantage over one-off authentication methods in that a user cannot log in initially and then allow somebody else to use the system because authentication is continuous.

Continuous authentication typically relies on the use of wearable devices, which are commonplace in immersive XR systems. Researchers have proposed various types of continuous authentication using wearable devices, including measuring in-ear sound waves [62] and pulsatile signals from a photoplethysmography (PPG) sensor [63]. These can also be combined with other techniques; for example, Ryu et al. [64] proposed a mutual authentication scheme for the Metaverse using biometric information, elliptic curve cryptography, and blockchain technology. However, while continuous authentication presents several advantages over traditional means, authentication accuracy and speed are factors that must be considered in this direction of research.

### 6.2. Automated Detection and Mitigation

The Metaverse will be an extremely large system with many simultaneous users and a huge amount of transactions occurring in real-time over the Internet. Hence, it will be infeasible for users, or even domain experts, to detect and deal with cybersecurity threats manually. This demands the development of automated methods to detect and mitigate potential cyber threats. As such, this will heavily rely on AI-driven cybersecurity to detect abnormal and malicious activities in the Metaverse in an automated manner.

This is a promising research direction because AI-driven cybersecurity techniques can be used in a variety of different Metaverse areas. For example, it can potentially be used to detect identity fraud and impersonations [40], to detect XR malware intended to cause physical harm [12,50,61] or exploit vulnerabilities [49], to monitor behavior that violates social norms and online abuse, to mitigate cybersickness by dynamically adjusting virtual world functionality [60], for behavior and biometric authentication [37,39,63], and many others.

### 6.3. Cybersecurity Awareness

In another research direction, researchers have shown that visualization technologies can be used for other purposes in cybersecurity. For instance, Alqahtani and Kavakli-Thorne [65] presented an AR game designed to increase awareness of cybersecurity issues in an entertaining manner. Visualization technologies can also be combined with digital twin technology to facilitate situational awareness of cybersecurity threats. The concept of a digital twin is a virtual representation of a physical real-world object in the virtual world. Böhm et al. [21] suggest that AR and digital twin technology can be used to enhance situation awareness for security professionals and domain experts through the direct connection of real-world objects in cyberspace, which can help facilitate the decision-making process when dealing with cyber threats.

## 7. Conclusions

Despite the amount of attention and investment in the Metaverse, the development of the Metaverse is still in its infancy. There are various cybersecurity issues that must be addressed before the Metaverse can truly be adopted in practice. Given the view that visualization technologies are a key component of the Metaverse, this visual aspect gives rise to emerging cybersecurity threats that have not previously received much attention. This survey presents an overview of the cybersecurity threats faced by the Metaverse in relation to visualization technologies. This paper also discusses existing work and promising open research directions in the development of countermeasures against such threats. 

## Figures and Tables

**Table 1:** Summary of recent surveys on the Metaverse and related technologies.

Category	Reference	Contribution
General	Cheng et al. [1]	Describes the positions of major tech companies and the requirements of the Metaverse.
	Park and Kim [3]	Discusses concepts and essential techniques for realizing the Metaverse.
Technology	Yang et al. [14]	Provides a survey of how blockchain and AI technologies can be fused with the Metaverse.
	Huang et al. [15]	Presents a survey on integrating building information modeling and blockchain technologies with the Metaverse.
	Fu et al. [16]	Reviews the role of blockchain and intelligent networking in providing immersive Metaverse experiences.
	Huynh-The et al. [17]	Investigates the role of AI and its integration in the development of the Metaverse.
Security	Wang et al. [4]	Presents a survey on the fundamentals, security, and privacy of the Metaverse.
	Fernandez and Hui [18]	Provides an overview of privacy, governance, and ethical design, in the development of the Metaverse.
	Di Pietro and Cresci [19]	Discusses several security and privacy issues, and risks in the context of the Metaverse.
	Odeleye et al. [20]	Creates a taxonomy of cybersecurity challenges faced in VR environments.
	Böhm et al. [21]	Systematizes knowledge on AR and digital twin technology and discusses how cybersecurity can benefit from them.
	De Guzman et al. [22]	Presents a systematic literature survey of security and privacy approaches in mixed reality (MR).

**Table 2:** An overview of visualization and cybersecurity issues and potential countermeasures.

Category	Issues	Countermeasures
Authentication and identity	Inference attacks [9,10,31,32,33,34]	XR authentication [35,36,37,38,39]
	Identity theft and impersonation attacks [40,41,42]	AI-driven detection [40,43,44,45,46]
Privacy issues	Location privacy [11,47]	Privacy strategies [47]
	Behavior privacy [47,48,49]	XR forensics [48,49,50]
	Video feed leakage [12,51]	
Social issues	Virtual spying and stalking [52]	Rules and system control [18,53,54]
	Virtual harassment and abuse [55,56,57,58]	Community-led governance [58,59]
Physical threats	Immersive attacks [12]	AI-driven detection [13]
	Cybersickness attacks [13]	Cybersickness mitigation [60]
	Puppetry and mismatch attacks [61]	

## Data Availability

Not applicable.
